# *g*-C_3_N_4_: Properties, Pore Modifications, and Photocatalytic Applications

**DOI:** 10.3390/nano12010121

**Published:** 2021-12-30

**Authors:** Jiaqi Dong, Yue Zhang, Muhammad Irfan Hussain, Wenjie Zhou, Yingzhi Chen, Lu-Ning Wang

**Affiliations:** 1 School of Materials Science and Engineering, University of Science and Technology Beijing, Beijing 100083, China; 13691129766@163.com (J.D.); irfanustb@163.com (M.I.H.); 2 Shunde Graduate School, University of Science and Technology Beijing, Foshan 528399, China; 13126595311@163.com (Y.Z.); zhouwenjie1459@163.com (W.Z.)

**Keywords:** *g*-C_3_N_4_, pore structure, template method, template-free method, photocatalysis

## Abstract

Graphitic carbon nitride (*g*-C_3_N_4_), as a polymeric semiconductor, is promising for ecological and economical photocatalytic applications because of its suitable electronic structures, together with the low cost, facile preparation, and metal-free feature. By modifying porous *g*-C_3_N_4_, its photoelectric behaviors could be facilitated with transport channels for photogenerated carriers, reactive substances, and abundant active sites for redox reactions, thus further improving photocatalytic performance. There are three types of methods to modify the pore structure of *g*-C_3_N_4_: hard-template method, soft-template method, and template-free method. Among them, the hard-template method may produce uniform and tunable pores, but requires toxic and environmentally hazardous chemicals to remove the template. In comparison, the soft templates could be removed at high temperatures during the preparation process without any additional steps. However, the soft-template method cannot strictly control the size and morphology of the pores, so prepared samples are not as orderly as the hard-template method. The template-free method does not involve any template, and the pore structure can be formed by designing precursors and exfoliation from bulk *g*-C_3_N_4_ (BCN). Without template support, there was no significant improvement in specific surface area (SSA). In this review, we first demonstrate the impact of pore structure on photoelectric performance. We then discuss pore modification methods, emphasizing comparison of their advantages and disadvantages. Each method’s changing trend and development direction is also summarized in combination with the commonly used functional modification methods. Furthermore, we introduce the application prospects of porous *g*-C_3_N_4_ in the subsequent studies. Overall, porous *g*-C_3_N_4_ as an excellent photocatalyst has a huge development space in photocatalysis in the future.

## 1. Introduction

In the last few decades, energy shortage and environmental remediation have become serious challenges to the long-term development of humanity. As an advanced green technology, semiconductor-based photocatalysis takes inexhaustible solar light as a driving force and a suitable semiconductor as a catalyst to cause photogenerated electrons and holes, and hence conducts photoredox reactions. Inorganic semiconductors (ISCs) are a group of widely used photocatalysts, such as TiO_2_, SnO_2_, ZnO, etc., which have the advantages of a high concentration of free charge carriers and high carrier mobility, and high physical and chemical stability [1]. However, they are usually limited by the narrow and fixed absorption spectrum [2], and the structural modification usually involves complicated fabrication processes or harsh conditions [3]. Compared with ISCs, organic semiconductors (OSCs) are characterized by structural diversity and synthetic functionality, and thus have easily tunable photoelectronic properties via structure/property engineering.

In the OSC family, conjugated polymers, *g*-C_3_N_4_ [4,5,6,7,8], polypyrrole (PPy) [9,10], polyaniline (PANI) [11,12], polyimide (PI) [13], poly (3-hexylthiophene) (P3HT) [14], polyhydroxybutyrate (PHB) [15,16], etc., have been extensively used for photocatalytic purposes due to their broad, molecular-level tuning of optoelectronic properties. Among them, *g*-C_3_N_4_ resembles graphene and has a unique two-dimensional (2D) delocalized conjugated structure, which is formed by an infinite extension of triazine ring (C_3_N_3_) or tri-s-triazine ring (C_6_N_7_) as basic structural units [17]. Such structural features endow *g*-C_3_N_4_ with relatively larger SSA and hence more active sites for charge separation and redox reaction than other polymer semiconductors. In addition, the strong covalent bond between the triazine ring in *g*-C_3_N_4_ leads to extraordinary chemical and thermal stability (up to 600 °C) compared to other polymorphs, and this stability is the highest in organic materials. Moreover, *g*-C_3_N_4_ has the moderate bandgap (E_g_) of 2.7 eV, as well as a conduction band (CB) and valence band (VB) position at −1.4 eV and 1.3 eV (vs. normal hydrogen electrode (NHE)), respectively, which could meet various redox reaction potentials by easily engineering its structure [18]. Therefore, *g*-C_3_N_4_ has found ample possibilities in different photocatalytic application fields, mainly including overall water splitting [19,20,21], CO_2_ reduction [22,23,24], degradation of organic pollutants [25], and antibacterial [26,27,28,29].

**Figure 1:**
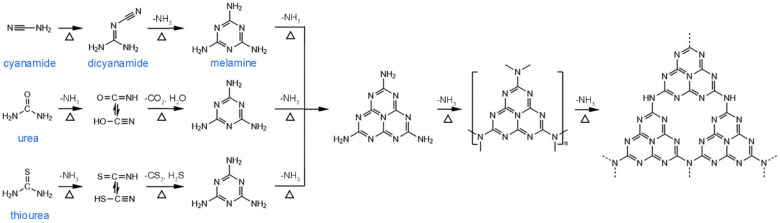
Thermal condensation process to prepare *g*-C_3_N_4_ from different precursors: cyanamide, dicyanamide, melamine, urea, and thiourea.

Despite these advantages, *g*-C_3_N_4_ still suffers from some drawbacks, such as narrow visible light response (~450 nm) and rapid recombination of photogenerated carriers, which limit their photocatalytic performance [30]. Numerous works have emerged to remedy the issues by applying second materials, such as doping metals [31,32] and non-metals [33,34], or combining with other semiconductors [35,36], adsorptive materials [37], and photosensitizers [38]. In addition, diverse synthetic pathways to obtain *g*-C_3_N_4_ structure have been employed in previous reports, for example, physical [39] or chemical vapor deposition [25,40], solvothermal method [41,42,43], solid-state reaction [44], and thermal nitridation [45]. Among the above strategies, thermal condensation of several low-cost nitrogen-rich precursors is facile and the most commonly used method. Figure 1 shows the thermal condensation process to prepare *g*-C_3_N_4_ from different precursors, including cyanamide [46], dicyanamide [47], melamine [48], urea [49], and thiourea [50]. However, the SSA of BCN is still limited for enhancing their photocatalytic performance. It has been demonstrated that morphology and porosity control of pristine *g*-C_3_N_4_ could help achieve higher SSA and a high number of active sites for improving optical absorption and prolonging the lifetime of the photogenerated charge carriers. Modifying the pore structure of *g*-C_3_N_4_ appears to be an effective way to improve the optoelectronic properties and photocatalytic performance. Regarding the pores, it is generally assumed that micropores (<2 nm) have the smallest pore size and thus the largest SSA, which allows them to have more active sites to absorb particles, such as electrons and ions. Mesopores (2–50 nm) are more conducive to the transport of reactants or solvents because they have a shorter void distance between pores. Besides, multiple light reflection inside mesopores enhances light utilization efficiency. Macropores (>50 nm) have better adsorption of organic macromolecule or gas molecules for their removal and product transportation. From there, pore size control toward a good balance between light absorption, reactant adsorption, and charge/mass transport is essential to high photon-to-electron conversion efficiency.

**Figure 2:**
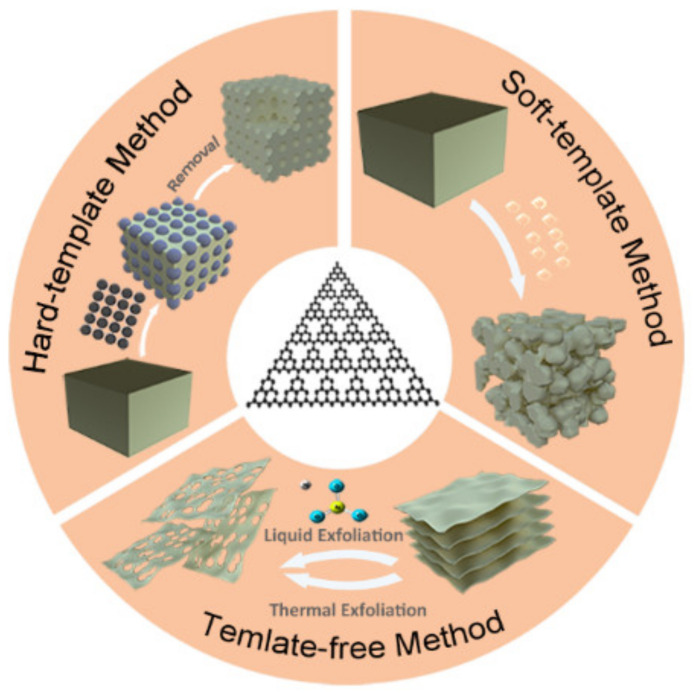
Pore modification methods over *g*-C_3_N_4_: hard-template method, soft-template method, template-free method.

Pore modification methods of *g*-C_3_N_4_ can be generally categorized into three types: hard-template method [51,52,53,54], soft-template method [55,56,57,58,59], and template-free method [60,61,62,63] (Figure 2). Hard template provides good support in yielding relatively regular pores that can act as a good channel for the migration of photogenerated charge. While the experimental operation process is relatively complicated because the templates must require an additional removal process. Soft-template method mainly introduces templates with diverse morphologies and can be easily decomposed during the condensation of *g*-C_3_N_4_. The template-free method does not involve any templates, which simplifies the experimental process to the greatest extent and reduces the cost. Using these methods, it is possible to control the pore volume, pore size, and SSA of the prepared porous *g*-C_3_N_4_ to modify the optoelectronic properties and photocatalytic function.

In this review, the pore structure-dependent properties are first introduced. Furthermore, we discuss three pore modification methods (hard-template method, soft-template method, template-free method) with their advantages and disadvantages, highlighting the effects of pore structure on the photoelectric properties and photocatalytic performance. Recent achievements of pore structure *g*-C_3_N_4_ in photocatalytic fields, including water splitting, CO_2_ reduction, and wastewater treatment, are then introduced. Finally, some challenges and prospects for the future development of porous *g*-C_3_N_4_-based photocatalysts are proposed.

## 2. Pore Structure/Properties Relationship

The pore structure in the material has a great influence on its electrochemical properties [64,65,66,67,68]. As discussed early, micro- and mesopores are important in creating a large accessible surface and interface areas for charge carrier generation, transfer, and transport, and macropores are favorable for mass transport.

Microporous materials have the smallest pore size and could attain the largest SSA, allowing them to expose more active sites to electrons and ions. Numerous studies have revealed that the micropores are crucial in activating O_2_ and other small molecules, such as H_2_O, by absorbing more electrons and ions. A microporous graphitic carbon with strictly regular porosity and diameter of 0.63–0.97 nm, has demonstrated ultra-high SSA of 1927.6 m^2^·g^−1^, giving a photocatalytic H_2_ evolution rate of 58.3 μmol·h^−1^·g^−1^ [69]. Metal-organic frameworks (MOFs), well known as porous coordination polymers, have been considered as ideal precursors for well-designed semiconductors with microporous structures. For example, Fe-based MOFs Fe-MIL-100 with a pore size of ~2 nm exhibited an SSA of 1203.36 m^2^·g^−1^, yielding a tetracycline (TC) degradation constant of 9 × 10^−3^ [70]. However, Fe-MIL-101 with a larger pore (25.74 nm) and lower SSA showed better photocatalytic performance (1.63 × 10^−2^). On one hand, the more micropores, the larger the SSA materials will get. On the other hand, the relationship between SSA and properties is not always positive. Because the void distance of micropores will be relatively longer, which is not conducive to the diffusion of reactants and the occurrence of reaction in terms of kinetics. Therefore, there is rare literature on microporous *g*-C_3_N_4_ in photocatalysis.

As for mesoporous materials, their pore size is larger than that of micropores materials and smaller than that of macropores materials. Generally, suppose the porous material is used as the catalyst carrier. In that case, the active metals are generally distributed in the microporous part, and the larger metal particles will be in the mesoporous. Compared with micropores materials, mesoporous are more conducive to the transport of reactants or solvents because they have a shorter void distance between pores. For example, Tian et al., presented a three-dimensional (3D) ultrathin mesoporous N doped *g*-C_3_N_4_ (UM3) with urea and melamine as precursors [71]. UM3 possessed the highest SSA (39.1 m^2^·g^−1^) with a pore diameter of 46.1 nm among all the samples. The UV-vis diffuse reflectance spectrometry (DRS) showed that UM3 has the lowest E_g_ of 2.47 eV, while BCNs is 2.70 eV. The narrower E_g_ allowed UM3 to increase the utilization ability of the solar spectrum. As for the result, the UM3 yielded an H_2_ evolution rate of 3579 μmol·h^−1^·g^−1^, which was almost 23 times higher than that of BCN (147 μmol·h^−1^·g^−1^).

On the other hand, macropores materials have better adsorption for liquids with high viscosity. However, the pore size of macropores is too large for ordinary reactants or solvents. Therefore, the pure macropores materials are not very practical. They can be useful for exposure to other kinds of pores [72]. In general, if a material has these three kinds of pores simultaneously, it can integrate the characteristics of the three kinds of pores materials and be more effective in its application. These types of materials are called hierarchically porous materials [73,74]. Li et al., synthesized macroscopic foam-like holey ultrathin *g*-C_3_N_4_ nanosheets (CNHS) with micro-, meso-, and macropores by thermal polycondensation of melamine [75]. The SSA of CNHS and BCN are 227.98 and 10.89 m^2^·g^−1^, respectively. Notably, the macropores in CNHS accounted for the maximum ratio of 52.1% for the total volume. Still, they accounted for the minimum ratio of 16.8% for SSA, while the mesopores in CNHS accounted for 46.7% of the total volume and the maximum ratio of 57.8% for the SSA. This meant that the macropores mainly serve for the exposure of the mesopores. As for the optical properties, the DRS showed a slight blue shift of the intrinsic absorption edge of CNHS. The E_g_ of the CNHS was 2.81 eV, larger than that of the BCN (2.70 eV). This may lead to a larger thermodynamic driving force in photocatalytic H_2_ evolution. Consequently, the H_2_ evolution rate of CNHS was 57.20 μmol·h^−1^, which was over 22 times larger than that of BCN. Herein, most of the porous *g*-C_3_N_4_ used are micropores, mesoporous, micro/mesoporous, or meso/microporous [76,77,78,79,80]. Similarly, Wei et al., prepared macropores (500–700 nm) and mesopores (2–20 nm) Zr-based MOF Zr_6_O_4_(OH)_4_(BDC)_12_ (UiO-66) to form advanced CdS/UiO-66 catalysts [81]. They found that the pore size impacted the spatial distribution of CdS nanoparticles: CdS tended to deposit on the external surface of mesoporous UiO-66, but spontaneously penetrated the large cavity of macroporous UiO-66 nanocage. By loading CdS, the photocatalytic reaction constant of macroporous CdS/UiO-66 over 4-nitroaniline reduction was three times higher than that of the mesoporous counterpart.

## 3. Pore Modifications over *g*-C_3_N_4_

### 3.1. Hard-Template Method

During hard-template method, the templates are mixed and coated with the precursors of *g*-C_3_N_4_ to generate pore structures according to the morphology of the templates. The existence of the templates provides good support for the sample. It makes the sample form a relatively regular pore structure, which provides good channels for the transmission of photoelectric charge. SiO_2_ nanostructure and SiO_2_ derivatives are mainly used in the hard-template method, which must be prepared in advance and removed by chemical reagents after the sample is formed [82,83,84]. The directly acquired salt templates simplify the experiment process, and importantly they could be removed with eco-friendly reagents.

#### 3.1.1. SiO_2_ as Template

Many studies have shown that a zero-dimensional (0D) SiO_2_ nanostructure is widely used as a hard template. For one, SiO_2_ has stable properties and suitable hardness, providing good support for samples. Second, the nanostructure of SiO_2_ is regular and fine-tunable. Therefore, pores of *g*-C_3_N_4_ are ordered in shape and tunable in diameter along with SiO_2_ in different sizes. Li et al., successfully synthesized porous C_3_N_4_ with dicyandiamide (DCDA) as precursors and SiO_2_ nanoparticles (SiO_2_ NPs) (7–40 nm) as hard templates [85]. In this study, DCDA and SiO_2_ were dispersed with deionized water and dried to obtain a solid mixture. Then, the solid mixture was calcined to obtain *g*-C_3_N_4_ containing SiO_2_. Finally, the sample was treated with 20% HF for four hours to obtain porous *g*-C_3_N_4_. The as-prepared samples had mesopores (5–40 nm) and a larger SSA of 109 m^2^·g^−1^, which was nearly 10 times of the BCN (11.89 m^2^·g^−1^). The larger SSA brought by the more porous structure increased the adsorption of RhB. The photocatalytic rhodamine B (RhB) degradation efficiency of porous C_3_N4 (100%, in 30 min) was higher than that of the BCN (62%, in 30 min) [86]. However, the pore size of *g*-C_3_N_4_ was non-uniform due to the replication of multisize SiO_2_, so there was no significant enhancement of light absorption.

**Figure 3:**
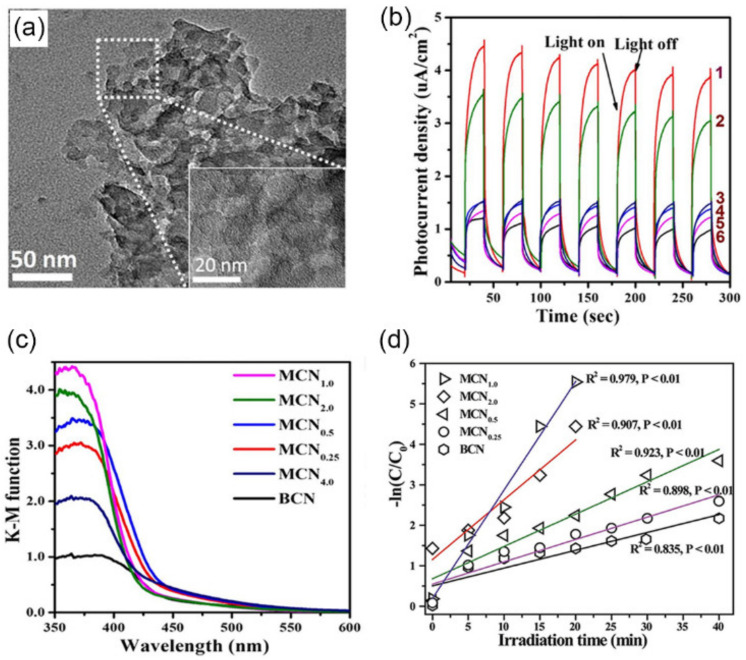
(**a**) Transmission electron microscope (TEM) images of MCN_1.0_; (**b**) photocurrent responses spectra of as-synthesized BCN and MCN_r_ samples (the numbers on each line correspond to 1: MCN_1.0_; 2: MCN_2.0_; 3: MCN_4.0_; 4: MCN_0.5_; 5: MCN_0.25_; and 6: BCN); (**c**) UV-vis DRS and (**d**) the rate constant for photocatalytic U(VI) reduction in BCN and MCN_r_ photocatalysts. Reprinted with permission from [87]. 2020 Elsevier.

The narrow scale range and appropriate content of SiO_2_ is important to the regularity of pore structure. In an example, SiO_2_ nanospheres with uniform size (~12 nm) were used as a hard template, which was mixed into precursor aminonitrile to prepare mesoporous *g*-C_3_N_4_ (MCN) [87]. According to the amounts of SiO_2_ (0.625, 1.25, 2.5, 5.0, and 10 g), the as-prepared samples were denoted as MCN_r_. The average pore sizes were uniformly near 12 nm for all samples, which could be regarded as a replica of the structure of the SiO_2_ spheres. Compared with BCN (9 m^2^·g^−1^), the SSA of MCN_r_ was significantly improved (23–191 m^2^·g^−1^). When a large amount of the template was applied, the speculation that stackable small C_3_N_4_ pieces could be formed, resulting in destroying the pore structure, was proved in the sharply decreased total pore volume of MCN_4.0_ (0.05 cm^3^·g^−1^) for other MCN_r_. Among all the samples, MCN_1.0_ had the largest SSA of ~191 m^2^·g^−1^ and total pore volume of ~0.52 cm^3^·g^−1^, respectively, which were 21.8 and 13.0-fold higher than that of BCN, respectively (Figure 3a). Due to the presence of regular mesopores and nanoscale pore walls, MCN_1.0_ photoelectric performance was remarkably improved. For one reason, MCN_1.0_ exhibited an upshift in the 400–600 nm for intensified light absorption (Figure 3b). Besides, charge separation of MCN_1.0_ also was enhanced, as proved by the highest photocurrent intensity (4.11 μA·cm^−2^), 4.0 times of BCN (1.03 μA·cm^−2^) (Figure 3c). Therefore, the reaction rate constant for photocatalytic U(VI) reduction in MCN_1.0_ (0.27 min^−1^) was 6.75 times that of BCN (0.04 min^−1^) (Figure 3d).

#### 3.1.2. SiO_2_ Derivatives as Templates

In addition to SiO_2_ nanostructures, some SiO_2_ derivatives with intrinsic pore structures are developed as hard templates, such as santa barbara amorphous-15 (SBA-15) [88,89,90,91,92,93], mesocellular silica foam (MCF) [94], fibrous silica (KCC-1) [95], mesoporous silica (MCM-41) [96]. Due to the regulated pore diameter, these hard templates were applied to synthesize highly ordered and large SSA mesoporous *g*-C_3_N_4_.

**Figure 4:**
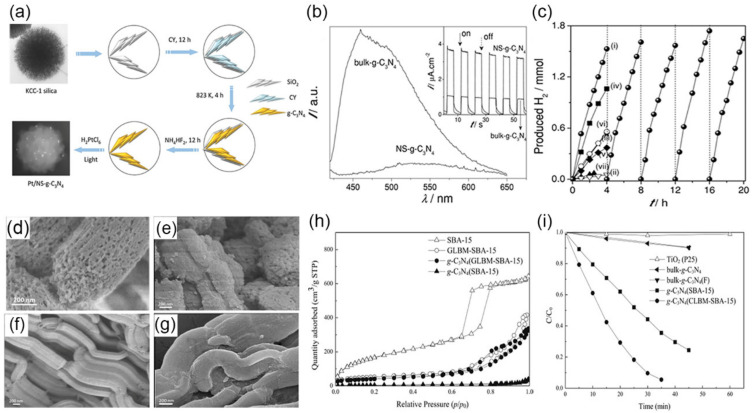
(**a**) Synthesis process of NS-*g*-C_3_N_4_; (**b**) PL spectra and photocurrent generation performance (inset) of NS-*g*-C_3_N_4_ and BCN; (**c**) Photocatalytic H2 evolution performance on (i) NS-*g*-C3N4, (ii) BCN, (iii) mesoporous *g*-C3N4, (iv) *g*-C3N4 hollow nanospheres, (v) *g*-C3N4 nanosheets, (vi) NS-*g*-C3N4 deformed by vigorous stirring in NH4HF2, (vii) NS-*g*-C3N4 deformed by grinding [98].; Scanning electron microscope (SEM) images of (**d**) CLBM-SBA-15, (**e**) *g*-C_3_N_4_(CLBM-SBA-15), (**f**) SBA-15, and (**g**) *g*-C_3_N_4_(SBA-15); (**h**) pore size distribution curves for SBA-15, *g*-C_3_N_4_(CLBM-SBA-15), *g*-C_3_N_4_(SBA-15), and CLBM-SBA-15; (**i**) photodegradation of MO on various *g*-C_3_N_4_ samples, Reprinted with permission from [83] 2015 Elsevier.

As the morphology of *g*-C_3_N_4_ depends on the initial silica matrices due to the peculiarities of the replication process, spherical mesostructured *g*-C_3_N_4_ could be obtained with 3D sponge-like foam MCF or nanospherical silica KCC-1. In one case, carbon nitride (CN) spheres with a uniform diameter (~4 μm) in size had a well-defined 3D foam mesopore structure by replicating spherical silica MCFs [97]. Such spherical CN materials possessed small (4 nm) and large mesopores (43 nm), a high SSA of ~550 m^2^·g^−1^, and a pore volume of 0.90 cm^3^·g^−1^. Through downsizing the template to a nanoscale, porous *g*-C_3_N_4_ nanostructure could be generated. For example, fibrous silica KCC-1 nanospheres were used as a template, yielding nanospherical *g*-C_3_N_4_ (NS-*g*-C_3_N_4_) by thermal-induced self-polymerization of cyanamide molecules [98]. After removing the template by NH_4_HF_2_, NS-*g*-C_3_N_4_ with a diameter of ~200 nm was faithfully replicated by the spherical morphologies of KCC-1 (Figure 4a). Besides, well-defined 2D sheets of KCC-1 supported the formation of interconnected 2D *g*-C_3_N_4_ nanosheets. The distribution of polymeric nanosheets endowed NS-*g*-C_3_N_4_ with a mesoporous structure (pore size: ~20 nm), featuring an SSA of 160 m^2^·g^−1^. Due to the multiple reflections of incident light within mesoporous architectures, the light-harvesting ability of NS-*g*-C_3_N_4_ was greatly enhanced to 590 nm from 450 nm of BCN. A similar structure-induced property change was demonstrated in charge transportation. 3D interconnecting nanosheets could significantly shorten the diffusion length of charge migration and promote electron re-localization on surface terminal sites. Therefore, the charge recombination was suppressed, as proven by strong photoluminescence (PL) quench (Figure 4b). The enhanced photocurrent could further suggest the improved charge separation (inset in Figure 4b). Therefore, NS-*g*-C_3_N_4_ exhibited a much-improved H_2_ evolution rate (574 μmol·h^−1^), far exceeding that of bulk-*g*-C_3_N_4_ (12.5 μmol·h^−1^) (Figure 4c).

Among mesoporous silica templates, well-ordered hexagonal SBA-15 exhibits uniform pore sizes (~30 nm) and large SSA (843 m^2^·g^−1^), which is a good choice to regulate pore distribution and enlarge SSA of *g*-C_3_N_4_ [99]. For example, a mesoporous CN prepared by SBA-15 as hard templates could achieve a superhigh SSA of 788 m^2^·g^−1^ and a large pore volume of 0.69 cm^3^·g^−1^ [100]. The conventional SBA-15 is synthesized by hydrothermal treatment in acidic media [101]. In one work, SBA-15 was first obtained using Pluronic P123 and tetraethyl orthosilicate in HCl solution under 150 °C heat treatment [102]. Then, by using this SBA-15, *g*-C_3_N_4_ with mesopore (~5.3 nm) was prepared, giving an SSA of 239 m^2^·g^−1^ and a pore volume of 0.34 cm^3^·g^−1^. The H_2_ evolution on the mesoporous *g*-C_3_N_4_ (~85 μmol·h^−1^) was about five times higher than BCN. Larger pore openings were essential for filling precursors to ensure complete replication of the pore structure of SBA-15. For example, researchers prepared a cross-linked bimodal SBA-15 (CLBM-SBA-15) with a larger pore opening size (44 nm) by decreasing the pH of the reaction solution to 7.5 [83]. Then, the mesoporous *g*-C_3_N_4_ samples were obtained by calcining the mixture of CLBM-SBA-15 or SBA-15 and precursor cyanamide. After that, the templates were removed with NH_4_HF_2_ solution, denoting the samples as *g*-C_3_N_4_(CLBM-SBA-15) or *g*-C_3_N_4_(SBA-15) (Figure 4d–g). Owing to the numerous pore openings of CLBM-SBA-15, *g*-C_3_N_4_(CLBM-SBA-15) had a pore size of 11–90 nm and a pore volume of 0.43 cm^3^·g^−1^. Compared to BCN and *g*-C_3_N_4_(SBA-15), *g*-C_3_N_4_(CLBM-SBA-15) had the largest SSA (145 m^2^·g^−1^), almost 8.5 and 20 times of *g*-C_3_N_4_(SBA-15) (17 m^2^·g^−1^) and BCN (7 m^2^·g^−1^), respectively (Figure 4h). The formation of the mesoporous structure could provide more surface-active sites for photocatalytic process and be helpful to the adsorption of organic compounds. Therefore, methyl orange (MO) degradation rate of *g*-C_3_N_4_(CLBM-SBA-15) reached 15.3 μg·min^−1^, which was 15.3 and 2 times than that of BCN and *g*-C_3_N_4_(SBA-15), respectively (Figure 4i).

#### 3.1.3. Salt as Templates

**Figure 5:**
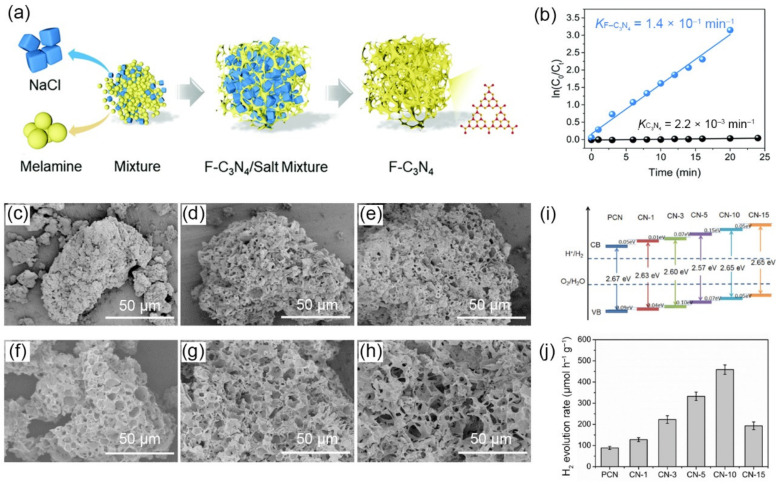
(**a**) Synthesis process of F-C_3_N_4_ by using melamine as precursor and NaCl as a template; (**b**) kinetic linear fitting curves of F-C_3_N_4_ and BCN, Reprinted with permission from [104]. 2020 Royal Society of Chemistry; (**c**) SEM images of CN, (**d**) CN-1, (**e**) CN-3, (**f**) CN-5, (**g**) CN-10, and (**h**) CN-15; (**i**) band structure and (**j**) photocatalytic H_2_ evolution rate of PCN and CN-xs, Reprinted with permission from [109]. 2019 Elsevier.

In recent years, several studies on using metal salts (e.g., Na_2_S_2_O_3_ [103], NaCl [104,105], KCl [106,107], ZnCl_2_ [108]) as hard templates have appeared. These salts are easily acquired, which could simplify the preparation process. During this method, the solid salts are dissolved with precursors in the solution, and then the solvent is removed by rotary evaporation or freeze-drying, yielding a powder mixture. After the calcination of a mixture, the samples were added into hydrochloric acid or deionized water for template removing, which is environmentally friendly. In one case, 3D porous foam-like *g*-C_3_N_4_ (F-C_3_N_4_) was prepared by thermal polycondensation of melamine with NaCl as templates (Figure 5a) [104]. F-C_3_N_4_ bared a macropore-dominated hierarchically porous structure (pore size-range of 0.1–1 μm, centered at 60 nm) with an SSA of 50.3 m^2^·g^−1^, which was 12-fold of BCN (4.2 m^2^·g^−1^). Electrochemical impedance spectroscopy (EIS) plots showed that the arc radius of F-C_3_N_4_ (~151 kΩ) was smaller than that of pristine *g*-C_3_N_4_ (~191 kΩ), indicating its higher separation efficiency of electron-hole pairs and charge transfer ability. Great adsorption ability to tetracycline molecules of macropores also facilitated the photocatalytic process, as it was observed that the removal efficiency of TC by F-C_3_N_4_ was 5.65% in the dark, while there was no obvious adsorption of TC by BCN. Thanks to the synthetic effect, 99.7% TC was degraded by F-C_3_N_4_ after light irradiation, and only 4.22% TC was removed by BCN (Figure 5b).

As a hard template, the number of added salts plays an important role in adjusting photoelectric properties by tuning the pore structure of *g*-C_3_N_4_. Besides, metal salts will cause element doing (e.g., Na^+^, K^+^, O) on porous *g*-C_3_N_4_ during heat treatment, influencing its band structure. For instance, Yang et al., fabricated a macrostructure *g*-C_3_N_4_ (pore size in a range of several hundred nanometers to several micrometers) through one-step calcination by using DCDA as a precursor and water-soluble NaCl cubes as the template, named CN-x (x, the molar ratio of NaCl/DCDA) [109]. After calcination, samples were washed with deionized water several times to remove the template. SEM images (Figure 5c–h) showed that the pore morphology of CN-x varied as the molar ratio of NaCl/DCDA changed. With the increment mass of NaCl, the number of holes was increased, and the size became larger, giving the higher SSA (up to 16.71 m^2^·g^−1^ of CN-10). When adding NaCl, the pore wall got thin because of closely assembled NaCl cubes, which destroyed the pore structure, leading to the decrease in SSA (11.23 m^2^·g^−1^ of CN-15). Except for pore modification, the introduction of salt caused Na^+^ doping, influencing band structure of CN-x samples, as proved by the VB and CB in XPS results (Figure 5i). Owing to high SSA and suitable band structure, CN-10 exhibited the best photocatalytic H_2_ evolution activity (459 μmol·h^−1^·g^−1^), about 5.2-fold of its bulk counterpart (Figure 5j).

In preparing photocatalyst experiments, the main role of a hard template is to construct and stabilize the pore structure. The pore structure can provide a channel for the transport of photogenerated electrons, promote the separation of electrons and holes, inhibit the recombination of electrons and holes, and thus enhance photocatalytic activity. Using SiO_2_ as hard templates can ensure the regularity of pore structure. Porous SiO_2_ derivative templates of different shapes endow *g*-C_3_N_4_ corresponding morphology, highly ordered pore distribution, as well as high SSA. However, the removal of SiO_2_ requires toxic and harmful etchants, such as HF or NH_4_HF_2_, which will do harm to the environment. Although using salt as a hard template can simplify the experiments without template prefabrication and avoid the usage of pollutant reagents, removing the hard template is still needed. Herein, researchers were seeking other convenient ways to produce pore structure in *g*-C_3_N_4_.

### 3.2. Soft-Template Method

The soft-template method is a facile and effective way to produce high surface area [46]. This method mainly introduces templates that can be removed with high temperature in the preparation process. The selection of soft templates is more varied than that of hard templates, and the morphology is diverse, including the various surfactants and bubble templates. Besides, some precursors can be directly used as soft templates. The key is that these soft templates would be dissolved or released during heat treatment, so the separate steps to remove the template are no longer needed [110].

#### 3.2.1. Surfactants as Templates

Surfactants are applied as a surface agent to the precursors, which form pores by their decomposition during thermal condensation of the precursors. This method requires that the surfactants have certain thermal and chemical stability in case of ahead decomposing. Besides, surfactant templates should be easily and completely decomposed to avoid affecting the electrical conductivity of *g*-C_3_N_4_. Commonly used surfactant soft templates include nonionic surfactants (e.g., Pluronic P-123 [111,112], Triton X-100 [46,113,114,115], Pluronic F-127 [116]) and ionic liquid [117,118,119].

**Figure 6:**
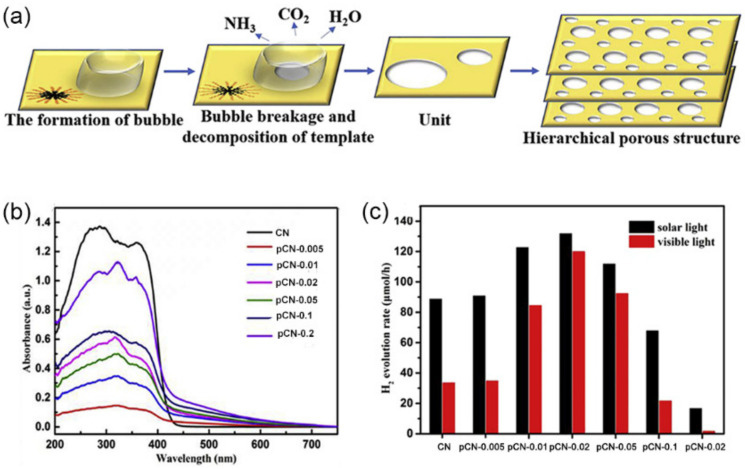
(**a**) Formation mechanism of highly porous pCN-X; (**b**) UV-vis DRS of CN and pCN-X (X = 0.005, 0.01, 0.02, 0.05, 0.1, 0.2); (**c**) photocatalytic H_2_ evolution rate of pCN-X under solar and visible light irradiation (λ ≥ 400 nm), Reprinted with permission from [122] 2017 Elsevier.

The assistance of nonionic surfactants as soft templates has improved the surface area and porosity of *g*-C_3_N_4_. A study has shown that mesoporous CN synthesized with Pluronic F-127, Pluronic P-123, and Triton X-100 reached higher SSA (145, 164, and 186 m^2^·g^−1^) than that of BCN (10 m^2^·g^−1^) [120]. In addition, their pore volume was 0.51, 0.55, and 0.6 cm^3^·g^−1^, far exceeding that of BNC (less than 0.1 cm^3^·g^−1^). It was demonstrated that ionic liquid, consisting of a hydrophilic ionic head group and a hydrophobic organic chain, can self-assemble into micelles like surfactants in the aqueous solution, which have been widely used to induce the porous materials [121]. Such a strategy can also be involved to construct porous structure *g*-C_3_N_4_ photocatalysts. The benchmark performance of urea tended to grow around the micelles due to the weak hydrogen bonding with ionic liquids (1-butyl-3-vinylimidazolium bromide) [122]. Meanwhile, the ionic liquid micelles could act as the center of the nucleation and growth of the *g*-C_3_N_4_ nanosheets. Thus, porous structures (pore size: ~85 nm) consisting of *g*-C_3_N_4_ nanosheets were prepared by the decomposing of ionic liquid micelles during the condensation of urea at a high temperature (Figure 6a), denoted pCN-X (X: the additive amount of ionic liquid). The calculated SSA and pore volume were 149.6 m^2^·g^−1^ and 0.796 cm^3^·g^−1^, respectively, which was about 3.6 and 4.6 times higher than those of pure CN (41.2 m^2^·g^−1^, 0.17 cm^3^·g^−1^). Owing to the multiple reflections of incident light within the porous structure, the pCN showed an extended light absorption in the range of 410–720 nm compared to 200–409 nm of BCN (Figure 6b). An outstanding H_2_ evolution rate (120 μmol·h^−1^) was achieved, about 3.6 times higher than BCN (Figure 6c).

The choice of the precursor is important because the interaction between the precursor and the template affects the degree of polymerization [120]. The strong interaction between the surfactant molecule and precursors causes incomplete condensation of the precursor and the high level of carbon residue. The heavily doping contents would offer more potential recombination sites of the semiconductors, reducing their photocatalytic performance. When using Pluronic P123 as a template and DCDA as a precursor, a mesoporous *g*-C_3_N_4_ yielded disordered pore systems and a high molar ratio of C/N (0.82–2.06) because of the strong hydrogen bonds between PEO blocks of Pluronic P123 and DCDA [46]. In general, the heavily carbon contents would offer more potential recombination sites of the semiconductors, which have a negative effect on the improvement of photocatalytic performance. Considering melamine is a less reactive precursor than DCDA, Yan et al., used melamine as a precursor and Pluronic P123 surfactant as a soft template to avoid the direct chemical reaction between them, giving worm-like mesoporous *g*-C_3_N_4_ (pore size: ~20 nm) [123]. During thermal treatment, CN polymerization and surfactant combustion were independent, so that prepared *g*-C_3_N_4_ had a minor amount of carbon dopants (C/N: 0.69) and possessed a higher SSA of 90 m^2^·g^−1^ (10 times that of pristine *g*-C_3_N_4_). Therefore, the sufficient light absorption extended to the maximum of the solar spectrum (up to 800 nm), which played an important role to enhance photocatalytic H_2_ evolution rate (148.2 μmol·h^−1^) with respect to the sample synthesized without using the surfactant (60.5 μmol·h^−1^).

#### 3.2.2. Bubbles as Templates

Bubble template refers to the gases released by the decomposition of pore modifiers, which can promote the formation of porous structures during the calcination of the precursor. A kind of bubble template, such as H_2_O and CO_2_, only helps with supporting holes and has no effect on the precursor molecule itself. A sucrose-mediated *g*-C_3_N_4_ with small mesopores (2.3–3.5 nm) formed by H_2_O and CO_2_ released from sucrose had an SSA of 121 m^2^·g^−1^, and showed an optimal H_2_ evolution rate of 107.8 μmol·h^−1^, which was 8.6 times higher than that of pristine *g*-C_3_N_4_ [124]. Similarly, using H_2_O and CO_2_ generated by acetic acid as a pore-forming template, the mesoporous *g*-C_3_N_4_ (pore size: 5–50 nm) with an SSA of 138 m^2^·g^−1^ had 13.75-times higher RhB degradation rate (2.2 mg·L^−1^·min^−1^) compared to BCN (0.16 mg·L^−1^·min^−1^) [125].

**Figure 7:**
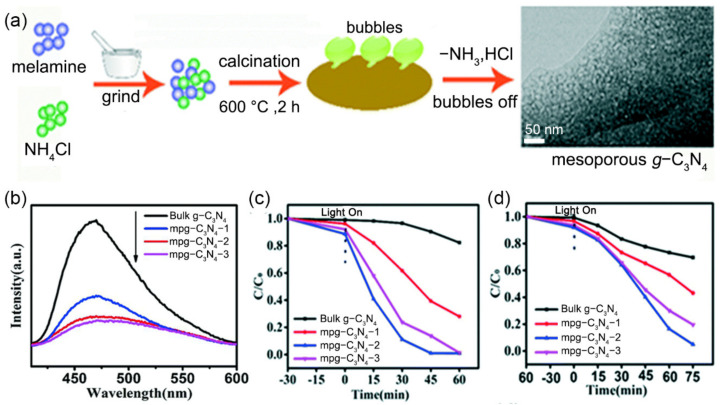
(**a**) Synthesis process of mesoporous *g*-C_3_N_4_; (**b**) PL spectra of the BCN and mesoporous mp*g*-C_3_N_4_ photocatalysts; visible light photodegradation rate constants of (**c**) RhB and (**d**) phenol of mp*g*-C_3_N_4_. Reprinted with permission from [126] 2011 Royal Society of Chemistry.

The inorganic ammonium salts (e.g., NHCl_4_, NH_4_HCO_3_) can decompose to produce the thermal gas flow to leave pores, and the generated gases, such as NH_3_ and HCl, would etch the s-triazine network structures, thus effectively promoting the production of pores and SSA. For example, taking NH_4_Cl as a bubble template and melamine as the precursor, mesoporous *g*-C_3_N_4_ (mp*g*-C_3_N_4_) with a pore size of ~13.9 nm exhibited 12-times higher SSA (195 m^2^·g^−1^) and 5-times higher pore volume (0.627 cm^3^·g^−1^) compared to BCN (17 m^2^·g^−1^; 0.127 cm^3^·g^−1^) (Figure 7a) [126]. Such extraordinarily higher SSA provided more potential reaction sites and channels for mass transfer to participate in photocatalytic reactions. Furthermore, the mesoporous *g*-C_3_N_4_ framework geometrically shortened the diffusion length of the photogenerated charge carriers to the surface, leading to a suppressed photogenerated charge recombination, as proved by a much weaker PL peak intensity (Figure 7b). This could contribute to the enhanced photocatalytic chemical reaction. As a result, the mp*g*-C_3_N_4_ sample exhibited the higher photodegradation activity for RhB (100%, 45 min) and phenol (96%, 75 min) than that of BCN (RhB: 20%, 45 min; phenol: 30%, 75 min) (Figure 7c,d).

**Figure 8:**
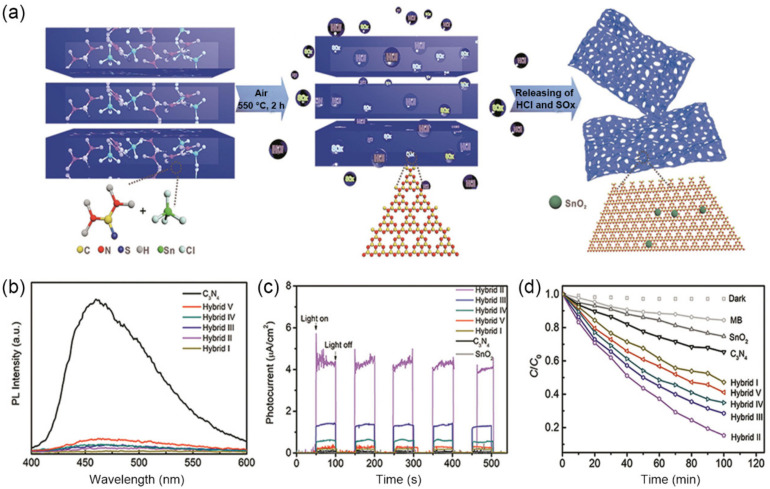
(**a**) Synthesis process of highly macroporous *g*-C_3_N_4_ system; (**b**) PL emission spectra and (**c**) photocurrent plots of the hybrids; (**d**) photocatalytic degradation of MB of the hybrids using under solar light, Reprinted with permission from [127] 2019 Elsevier.

Except for gas release to modify the pore structure, decomposition co-products of soft templates could couple with *g*-C_3_N_4_ to create charge transfer interfaces. For instance, Chen et al., utilized SnCl_4_ as a pore modifier and thiourea as precursor, obtaining macroporous *g*-C_3_N_4_/SnO_2_ nanohybrids (Figure 8a) [127]. During the synthesis process, SnCl_4_ was hydrolyzed to hydrous SnO_2_ and HCl. SnO_2_ was coupled with *g*-C_3_N_4_ to yield *g*-C_3_N_4_/SnO_2_ hybrids. At the same time, the released gaseous HCl acted both as an erosive soft template to tune the pore formation, giving the hybrids a pore size of 100–430 nm, a higher SSA of 44.3 m^2^·g^−1^ (6.5 times of BCN), and a higher pore volume of 2.638 cm^3^·g^−1^ (only 0.875 cm^3^·g^−1^ for BCN). As the continuous macropore channels favored light penetration into the solid samples and the larger volume of moderate macropores led to deeper optical penetration, the light absorption range was extended to 800 nm. Furthermore, because of the larger contacted area between *g*-C_3_N_4_ and SnO_2_, efficient photoinduced electron transfer across the interface was observed, as suggested by ~90% PL quenching (Figure 8b) and remarkably enhanced photocurrent generation (Figure 8c). As the result of macropore structure and the heterojunction, the MB degradation rate of macropores *g*-C_3_N_4_/SnO_2_ nanohybrid reached 98% in 100 min, which was 2.4 times higher than that of BCN (Figure 8d).

#### 3.2.3. *g*-C_3_N_4_ Precursors as Templates

Copolymerization is one of the most common synthetic approaches to preparing CN materials with extended light absorption [128,129,130,131]. In this technique, a mixture of two or more small organic monomers, obtained by grinding, ball-milling, or dissolution in common solvents, reacts at a high temperature, yielding CN materials [132]. Inspired by copolymerization, the choice of soft template is gradually shifting to the precursor itself without introducing other elements. The research about using two kinds of precursors to prepare the porous *g*-C_3_N_4_ increases gradually. One of the precursors is used as the template to form the pore structure, while the other acts as the main precursor of *g*-C_3_N_4_ matrix.

As the direct polymerization of urea can produce porous *g*-C_3_N_4_ without tailoring the reaction pressure and atmosphere, urea usually serves as a natural pore-forming agent [133]. For instance, porous *g*-C_3_N_4_ with a surface area of 60 m^2^·g^−1^ has been produced on a large scale by pyrolysis of urea and DCDA in air [134]. The rate content of optimum porous *g*-C_3_N_4_ for phenol decomposition was 0.039 h^−1^, which was 2.8 times as high as BCN. In another work, melamine was pre-treated in thiourea solution, and then the modified precursor samples were calcined to obtain porous, thin *g*-C_3_N_4_ nanosheets whose SSA increased from 9.0 to 44.2 m^2^·g^−1^ [135]. A 3.3-fold higher photocatalytic performance for H_2_ evolution (99.1 μmol·h^−1^) was achieved, in comparison with pristine one (29.7 μmol·h^−1^). Thiourea has the similar feature as urea, and was consequently used as a soft template. For example, thiourea was decomposed into gas bubbles during calcination, thereby inducing pore structure when copolymerized with DCDA [136]. This porous *g*-C_3_N_4_ with an average pore size of 3.7 nm, resulted in a 3.4-fold enhancement of SSA (46.4 m^2^·g^−1^) and an enhanced photocatalytic rate in the photodegradation of MB (0.146 h^−1^) with respect to the BCN (0.0430 h^−1^).

**Figure 9:**
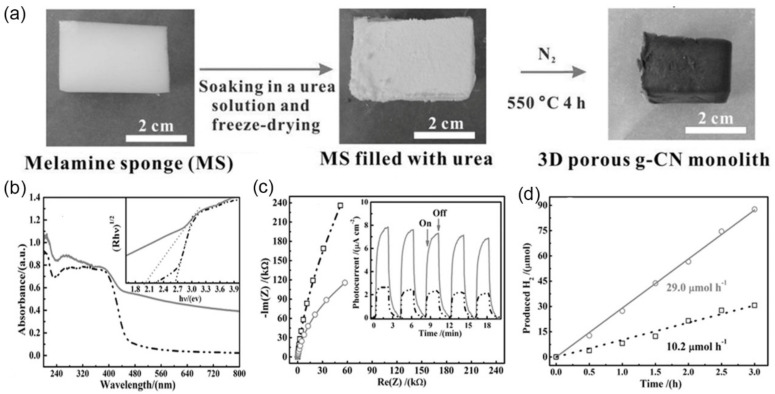
(**a**) Synthesis process of macroscopic 3D PCNM; (**b**) photocatalytic activity of H_2_ evolution; (**c**) EIS plots (inset: photocurrent-time dependence); and (**d**) DRS spectrum of PCNM (solid line) and the *g*-C_3_N_4_ powder (dotted line), Reprinted with permission from [30] 2015 John Wiley and Sons.

Besides forming pores by generating gas bubbles, heating-induced shrinking of the precursor framework can also help with the pore structure. Taking melamine sponge as soft template and urea as precursors, macroscopic 3D porous *g*-C_3_N_4_ monolith (PCNM) was synthesized with a pore diameter of 200–400 nm, SSA of 78 m^2^·g^−1^, and pore volume 0.76 cm^3^·g^−1^ [30]. Illustration of the preparation of a macroscopic 3D PCNM as shown in Figure 9a. Owing to the multiple reflections of incident light within the interconnected network of porous *g*-CN nanosheets, PCNM showed significantly improved light-harvesting ability above 450 nm in the optical spectrum compared to the powdered *g*-CN (Figure 9b). Furthermore, the 3D interconnected network of porous nanosheets ensured sufficient mass transportation. It shortened the diffusion length of charge migration, thus facilitating electron re-localization on surface edge sites to hinder the charge recombination, which could be suggested as a much smaller arc radius in EIS results (Figure 9c) and 2.5-fold increased photocurrent density (inset in Figure 9c). The prepared mesoporous *g*-C_3_N_4_ had a rate of H_2_ evolution of 29.0 μmol·h^−1^, which was 2.84 times that of ordinary *g*-C_3_N_4_ powder (Figure 9d).

There are several advantages of soft-template method. Firstly, the selection of soft templates is very rich and diverse. It makes the adjustment of sample structure more flexible, and the morphology of the pore structure can be adjusted by modifying the experimental design and preparation conditions. Secondly, the soft-template method can simplify the experimental process by reducing the removing step. Thereby, toxic and harmful reagents are avoided in the preparation process, which has less harm to the environment. Thirdly, the resulting lamellar and channel structures are conducive to light capture, improving light utilization and producing more photogenerated carriers. The disadvantage of the soft-template method is that it cannot always strictly control the size and morphology of the pores, so that prepared samples are not as orderly as the hard-template method. There are phenomena of interlayer collapse, interlayer stacking, and molecular polymerization, which hinder electron transmission. In addition, some soft templates have self-assembly behavior, which makes the reaction insufficient and leads to the failure of reaching the ideal SSA. Furthermore, some by-products will remain on the structure, cover the active sites, and affect the catalytic efficiency. Therefore, developing a green synthesis route to obtain PCN with high performances by soft-template methods remains challenging.

### 3.3. Template-Free Method

Template-free method refers to the construction process of pore structure by only one kind of precursor without involving a second template. According to literature research, there are mainly two ways to achieve template-free modification of pore structure: pre-treatment and a top-down approach. Pre-etching means that the pore of *g*-C_3_N_4_ is formed by condensation of hydro- or acid-treating precursors. Recently, thermal condensation of supramolecular precursors has emerged as a physiochemical approach to form the pore structure of *g*-C_3_N_4_, and the top-down approach is an exfoliation technique, especially for the synthesizing of porous *g*-C_3_N_4_ sheets from BCN. The template-free method could eliminate the step of template preparation, simplify the experimental process from sample preparation to the greatest extent, and reduce the cost at the same time.

#### 3.3.1. Pre-Etching of *g*-C_3_N_4_ Precursors

Porous *g*-C_3_N_4_ can be obtained via direct thermal calcination of the pre-treated precursors [137,138,139]. Hydrothermal pre-treatment is the most simple, economical, and convenient way, by which the pore structure could be formed without changing the crystal structure or chemical structure of precursors. For instance, porous *g*-C_3_N_4_ was produced through hydrothermal-treated cyanamide’s thermal polymerization [138]. Specifically, cyanamide solution was first heated at 150 °C, and then as-prepared white precursor was heated at 550 °C for polymerization. Many uniform pores (~30 nm) on *g*-C_3_N_4_ surface were driven by the etching effect of ammonia gas released during the hydrothermal pre-treatment process. The as-prepared porous *g*-C_3_N_4_ possessed an increased SSA (18.1 m^2^·g^−1^) in comparison to BCN (6.6 m^2^·g^−1^), and 7.2-fold higher photocatalytic H_2_ evolution rates (958 μmol·h^−1^·g^−1^) than that of BCN.

**Figure 10:**
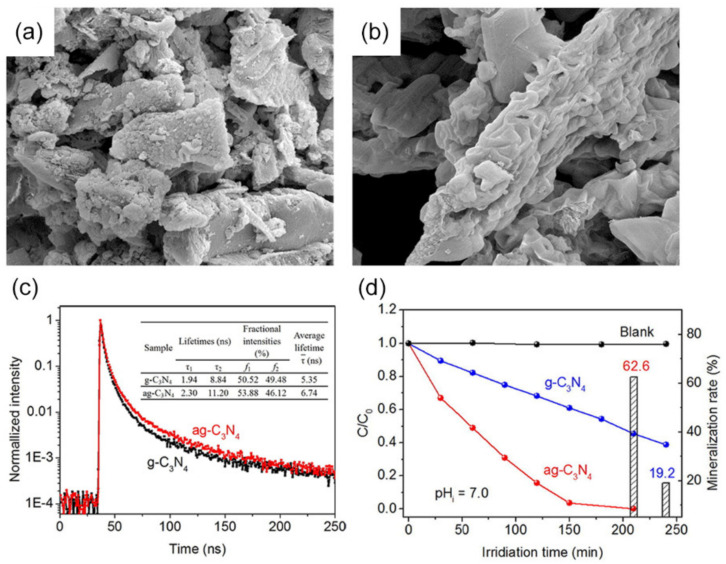
SEM images of (**a**) pristine *g*-C_3_N_4_ and (**b**) a*g*-C_3_N_4_; (**c**) time-resolved fluorescence decay spectra, and (inset) two-exponential analysis results of *g*-C_3_N_4_ and a*g*-C_3_N_4_; (**d**) comparison of PCD efficiency and mineralization rate of RhB by *g*-C_3_N_4_ and a*g*-C_3_N_4_ [144]. Reprinted with permission from [144] 2014 Elsevier.

A bottom-up acidification strategy refers to direct calcination of pre-formed precursor by hydrothermal treating in diluted acid solution. Previously reported works suggested that the acid, such as HCl, H_2_SO_4_ [140,141], H_3_PO_3_ [142], would act as an etching agent for nitrogen-rich precursor molecules, which contribute to the formation of pores during the polymerization of the acidified precursors. For example, *g*-C_3_N_4_ with a thin, porous platelet-like structure (p-*g*-C_3_N_4_) was fabricated via the polymerization of HCl-acidified melamine [143]. The *g*-C_3_N_4_ derived from HCl-acidified melamine exhibited a larger SSA (69.0 m^2^·g^−1^) than that of *g*-C_3_N_4_ derived from pure melamine (17 m^2^·g^−1^). The degradation constants of p-*g*-C_3_N_4_ were calculated to be 0.131 min^−1^, which was 9.4 times higher than *g*-C_3_N_4_. Similarly, Zhang et al., first treated the precursor melamine with HCl and alcohol and used it to prepare *g*-C_3_N_4_ photocatalyst (a*g*-C_3_N_4_) with a mesopore structure (pore size: 30.3 nm) (Figure 10a,b) [144]. The SSA and pore volume of a*g*-C_3_N_4_ (26.2 m^2^·g^−1^; 0.121 cm^3^·g^−1^) was higher than that of *g*-C_3_N_4_ (12.7 m^2^·g^−1^; 0.049 cm^3^·g^−1^), respectively. More active sites provided by a higher SSA facilitate the increase in active species for photocatalytic reaction, as proven by the increased fraction of the shorter-range charge carriers of a*g*-C_3_N_4_ (50.52%) for *g*-C_3_N_4_ (53.88%) (Figure 10c). In the presence of a*g*-C_3_N_4_, the RhB degradation efficiency rate was 99.9%, while with the catalysis by *g*-C_3_N_4_, the RhB degradation efficiency was only 62.6% (Figure 10d).

#### 3.3.2. Supramolecular Precursors

Recently, tuning porosity in CN through alteration supramolecular precursors has emerged entirely to be a new physiochemical approach [145]. In this approach, precursor monomers interact by non-covalent interactions (hydrogen bonding, π-π stacking, electrostatic interactions, etc.), forming a versatile self-assembled supramolecular in a given solvent. Designing the arrangement of the precursor monomers prior to the calcination leads to good control of the final *g*-C_3_N_4_ morphology and porosity, photophysical, and catalytic properties.

From a strengthened perspective, hydrogen bonds were mainly used to manipulate the monomer sequence designs, due to their exceptional strength and abundant donor and acceptor sites. A typical example is cyanuric acid-melamine supramolecular. It has been reported that cyanuric acid would be capsulated by melamine through hydrogen-bonding interaction and prohibit the polymerization of melamine to a certain extent. In one work, a porous *g*-C_3_N_4_ (p-CN) with a 3D hierarchical framework was obtained by thermal condensation of cyanuric acid-melamine supramolecular, featuring a 3.6-times larger SSA (35.6 m^2^·g^−1^) than that of BCN [146]. Besides, the hierarchical porous structure improved light-harvesting capacity and provided more active sites for photocatalytic reactions. As a result, p-CN showed an increased H_2_ evolution rate (68.5 μmol·h^−1^), which was approximately 4.8 times than that of BCN (14.3 μmol·h^−1^). In addition to the widespread utilization of the cyanuric acid-melamine supramolecular, the diversity of supramolecular structures formed by the self-assembly of the precursors has been reported to modify the final CN properties for an enhanced photoactivity, such as cyanuric acid-melamine-urea [147], melamine-urea [148], and dicyandiamide-urea [149].

**Figure 11:**
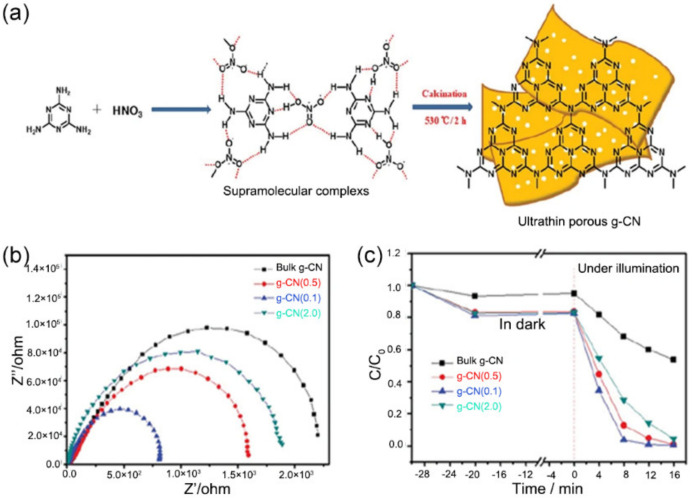
(**a**) Synthesis process of ultrathin porous *g*-CN nanosheets; (**b**) EIS Nyquist plots; and (**c**) photocatalytic RhB degradation rate constant of *g*-CN(x) and BCN, Reprinted with permission from [150]. 2019 Springer Nature.

Solvents used in the aggregation of supramolecular assemblies are key for the pore structure of *g*-C_3_N_4_. Solvents can serve as building blocks in the formation of supramolecular assemblies due to their hydrogen bonding sites. As an example, Zhu et al., obtained ultrathin *g*-C_3_N_4_ nanosheets (*g*-CN(*x*), *x* = 0.5, 1.0, 2.0 presented the concentration of HNO_3_) by calcining the HNO_3_-treated melamine supramolecular complexes (Figure 11a) [150]. The melamine supramolecular complexes were prepared by putting melamine in 1.0 mol·L^−1^ nitric acid, during which the NH…O (N) hydrogen-bonded supramolecular complexes formed through the interaction between melamine and HNO_3_. The broken hydrogen bond made *g*-CN(1.0) samples possess mesoporous/macroporous structures with a pore diameter of 10–100 nm. Besides, the SSA of *g*-CN(1.0) was 59.2 cm^2^·g^−1^, which was higher than bulk *g*-CN (15.6 cm^2^·g^−1^). Additionally, optimized ultrathin nanosheets with a porous structure are favorable for a photogenerated carrier effective transfer and separation, as suggested by a smaller arc radius of *g*-CN(1.0) on the EIS plots (Figure 11b), compared with BCN. As a result, the RhB degradation efficiency of *g*-CN(1.0) (0.352 min^−1^) was almost 10 times higher than that of the BCN (Figure 11c). Inspired by traditional liquid-liquid interfacial polymerization [151], interfacial supramolecular organization on two non-miscible solvents emerged in the modification of CN structure [152,153]. The choice of solvents plays an important role in the formation of supramolecular assembly at the interface of the solvents. For example, Dolai et al., employed water/chloroform interface to pre-organize precursor monomers (cyanuric acid and melamine), and added picric acid to tailor supramolecular framework structure so as to obtain the final CN porosity [154]. As the content of picric acid was increased, the tube-like structure of *g*-C_3_N_4_ gradually became more perforated, giving a sponge-like structures. An impressive enhancement of the SSA was obtained from 54 to 106 m^2^·g^−1^.

Recently, the utilization of highly ordered supramolecular single crystals based on CN monomers has been exploited as an effective approach to control the properties of CN materials [155,156]. Barrio et al., synthesized melaminium chloride hemihydrate single crystals, where protonated melaminium were connected by Cl^−^ and H_2_O [157]. By using such supramolecular single crystals as a precursor, the obtained needle-like porous CN nanostructure had an SSA of 185 m^2^·g^−1^, and an increased visible light absorption capacity in range of 450–600 nm. Besides, triazine-based cocrystals composed of two types of molecular units by non-covalent interactions, such as acetoguana-mine-melamine [158] and terephthalic acid-melamine [159], were selected as precursors for porous CN via pyrolysis. MOFs have emerged as a new type of precursors for porous carbons-based material and metal/carbon composites [160,161]. Due to the highly ordered crystalline structures, MOFs would be good candidates for use as sacrificial templates and precursors to give porous *g*-C_3_N_4_ [162]. Moreover, metal nodes transformed to metallic oxide under proper thermal condensation conditions, which would be further enhanced by forming heterojunction with *g*-C_3_N_4_. In one case, Wang et al., prepared porous nanorod-like *g*-C_3_N_4_/CuO composites by using a copper-melamine supramolecular framework as a precursor [163]. The nanorods had a pore size of about 20–50 nm, with the SSA of 15.37 m^2^·g^−1^, which was about three times higher than pure *g*-C_3_N_4_ (5.26 m^2^·g^−1^). Almost 94% of the RhB was degraded by *g*-C_3_N_4_/CuO under 20 min visible-light irradiation, while only 12% and 5% of RhB was degraded by *g*-C_3_N_4_ and CuO, respectively.

#### 3.3.3. Top-Down Approach

Because of the weak van der Waals force between layers in BCN frameworks, 2D porous *g*-C_3_N_4_ nanosheets were usually prepared by exfoliation from BCN. Such a pore-forming method was called the top-down approach. Recently, liquid exfoliation [164] or thermal exfoliation [165] strategies have also been employed to yield porous *g*-C_3_N_4_ nanosheets.

**Figure 12:**
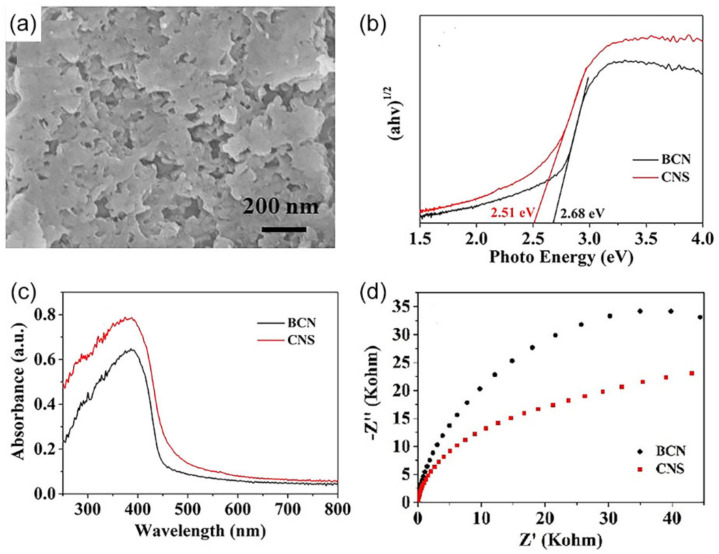
(**a**) SEM image of CNS; (**b**) estimated bandgaps, (**c**) UV-vis DRS, and (**d**) EIS plots of the BCN and CNS, Reprinted with permission from [168] 2021 Elsevier.

There are two main liquid exfoliation techniques for preparing porous *g*-C_3_N_4_ nanosheets: ion intercalation and ultrasound assist [166]. In one work, porous *g*-C_3_N_4_ nanosheets were obtained by lithium chloride ions in situ intercalating bulk materials in the thermal polycondensation process and followed by liquid exfoliation in water [167]. The nanosheets with 2–3 nm thickness and high-density in-plane pores with 2–3 nm gave a higher SSA of 186.3 m^2^·g^−^^1^ compared to BCN (9.8 m^2^·g^−1^). An enhanced H_2_ evolution performance for nanosheets (107.84 μmol·h^−1^) was observed, which was more than six times higher than that of bulk one (16.31 μmol·h^−1^). A more recent strategy for liquid exfoliation is to expose the BCN to ultrasonic waves in a solvent. Mesoporous *g*-C_3_N_4_ nanosheets (CNS) were fabricated by ultrasonically exfoliating the BCN [168]. SEM images are shown in Figure 12a. The as-prepared *g*-C_3_N_4_ nanosheets presented a higher SSA (55.41 m^2^·g^−1^) and a larger mesoporous volume (0.216 cm^3^·g^−1^) in comparison with the bulk counterpart (SSA: 7.61 m^2^·g^−1^; pore volume: 0.055 cm^3^·g^−1^). The resulting narrow bandgap (2.51 eV) (Figure 12b), wide light response (250–800 nm) (Figure 12c), and lower charge transfer resistance (Figure 12d) all contributed to a 3.3-fold photocatalytic H_2_ evolution rate of mesoporous *g*-C_3_N_4_ (97.6 μmol·g^−1^·h^−1^), compared to that of the BCN (29.8 μmol·g^−1^·h^−1^).

**Figure 13:**
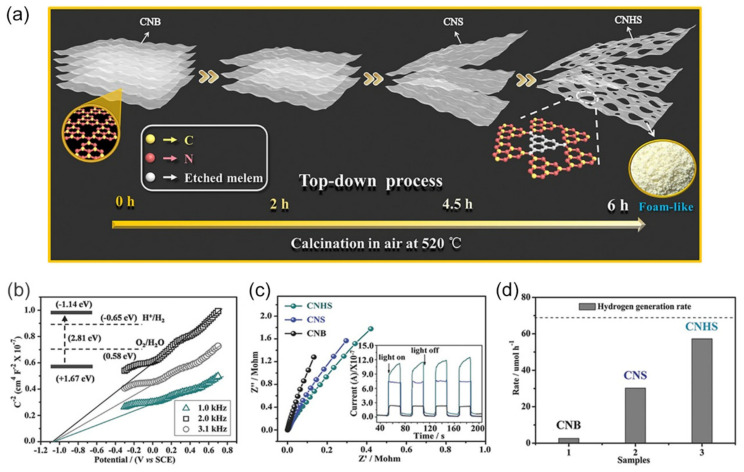
(**a**) Synthesis process of foam-like CNHS by top-down strategy; (**b**) Mott–Schottky plots with various frequencies of CNHS; (**c**) EIS plots (inset: transient photocurrent response) and (**d**) photocatalytic H_2_ evolution rate of CNB and CNHS, Reprinted with permission from [75] 2016 John Wiley and Sons.

It has been found that BCN can initially be thermally oxidized into nanosheets by overcoming the weak van der Waals force between layers [169]. Moreover, during the heating process, some tri-s-triazine units consisting of C-N bonds could also be “etched”, giving rise to the pore of *g*-C_3_N_4_ [170]. By increasing the calcination time, *g*-C_3_N_4_ with various pore sizes could be obtained by further thermal oxidation. Li and coworkers fabricated holey ultrathin *g*-C_3_N_4_ nanosheets (CNHS) (thickness: 9.2 nm) with abundant micro- (1–2 nm), meso- (2–50 nm), and macropores (50–100 nm) by thermal exfoliation of bulk *g*-C_3_N_4_ (CNB) at 520 °C for a continuous 6 h (Figure 13a) [75]. The SSA of CNHS was 277.98 m^2^·g^−1^, almost 26 times higher than that of CNB (10.89 m^2^·g^−1^). Compared to CNB, the CB potential of CNHS upshifted about 0.25 V (Figure 13b), which might lead to a larger thermodynamic driving force in photocatalytic hydrogen production. This larger bandgap could ascribe to the quantum confinement effect and the existence of in-plane holes, which decreased the conjugation system of *g*-C_3_N_4_. Moreover, the large number of in-plane holes greatly facilitated the mass transfer and improved photogenerated charge mobility, as suggested by smaller charge transfer resistance (Figure 13c) and higher photocurrent response (inset in Figure 13c) CNHS. Given the above, CNHS exhibited 22.24 times higher H_2_ evolution rate of 57.20 μmol·h^−1^, than that of CNB (2.57 μmol·h^−1^) (Figure 13d).

The advantage of the template-free method is that it can produce the pore structure by pre-treating precursors or exfoliating BCN. This can greatly reduce the consumption of raw materials and avoid using toxic and harmful reagents. Besides, there is no template residue in the preparation process, thus avoiding the negative impact caused by the existence of the template to the greatest extent. The biggest disadvantage of the template-free method is that its porous structure is formed by a natural reaction, so it is difficult to regulate the pore volume, pore diameter, and SSA of the samples. Secondly, due to the lack of template participation, *g*-C_3_N_4_ lacks effective support. Consequently, the phenomenon of molecular aggregation and interlayer stacking of samples prepared by a template-free method is common, resulting in unsatisfactory SSA.

Achievements of previous studies on pore modification of *g*-C_3_N_4_ have been concluded in Table 1. As discussed earlier, the pore modification method results in different structure regularity and SSA of the samples, arranged as follows: hard-template method > soft-template method > template-free method. This result is mainly because the different pore-support abilities of templates. The hard-template method requires a template removal step, which is inevitable and may use toxic and harmful reagents, so the hard-template method is gradually transitioning to the soft-template method or template-free method. However, the soft-template method has the problem of template residue covering the active site, which reduces catalytic efficiency. The pore structure formed by the template-free method is not easy to adjust and control, and interlayer stacking makes the SSA less than ideal. Therefore, specific strategies towards each pore structure modification have been studied. In the above cases, the sample prepared by the hard-template method has a higher SSA, but it increases the probability of recombination of electron holes. Therefore, energy band regulation is needed: different energy levels of other substances are used to form potential differences with the energy levels of *g*-C_3_N_4_ itself to promote electron transfer, thus greatly inhibiting the recombination of electrons and holes. The template support of the soft-template method is weak, the SSA of samples obtained by this method is smaller than that of a hard-template method, so that there are fewer active sites. Therefore, the precursors and soft templates should be reasonably selected. The special 3D structure should be combined to enhance the capture of light, to improve the utilization of light, and increase the number of photogenerated carriers. Template-free method used no template support during the process. The pore structure is mainly from chemical reaction and bubble escape, which leads to the difficulty to control the structure, prone to interlayer collapse and other phenomena, covering the active site. Therefore, it is necessary to use element doping to improve the electron transmission rate and increase the photoelectric current. The modification means that the three preparation methods are interrelated, and the effect is different, which should be selected according to the morphology and photoelectric properties of the sample.

## 4. Application

*g*-C_3_N_4_ possesses a moderate bandgap of 2.7 eV, with the CB and VB positions respectively at −1.4 eV and 1.3 eV (vs. NHE). Such band structure enables *g*-C_3_N_4_ to generate the active free radicals, photogenerated electrons, and holes with strong oxidization and reducibility for various photocatalytic applications. In coping with energy crises, *g*-C_3_N_4_ can split water into H_2_ and O_2_ and reduce CO_2_ into energy rich compounds. For environmental remediation, it can degrade several organic and inorganic pollutants into harmless substances. As discussed earlier, the photocatalytic efficiency of porous *g*-C_3_N_4_ has been improved compared with pristine *g*-C_3_N_4_. Based on tuning the pore structure of *g*-C_3_N_4_, researchers have carried out various modification strategies for enhancing photocatalytic efficiency to reach the desired effect, such as structure optimization, vacancies modify, element dope, and heterostructure construct. In view of this, the research progress of modified porous *g*-C_3_N_4_ in the field of different photocatalytic application are discussed in the following section.

### 4.1. Water Splitting

Photocatalytic water splitting to produce H_2_ and O_2_ is an attractive approach for the development of clean and renewable energy development. To realize water splitting, the CB of semiconductor photocatalysts must be more negative than the reduction potential of H+/H_2_ (0 V vs. NHE), and VB must be more positive than the oxidation potential of H_2_O/O_2_ (1.23 V vs. NHE). The position CB and VB of *g*-C_3_N_4_ at −1.4 and 1.3 eV, respectively, are highly suitable for carrying out the given redox reaction abilities. Besides, *g*-C_3_N_4_ with the bandgap of 2.7 eV exceed the free energy (1.23 eV) of water splitting under light irradiation. The excited electrons can cause the H^+^ reduction reaction to evolute H_2_, while the holes can cause OH^−^ oxidation reaction to form O_2_. Thus, *g*-C_3_N_4_ has the ability of overall water splitting to obtain H_2_ and O_2_. Significant achievements have been obtained on the porous *g*-C_3_N_4_ based photocatalysts for water splitting activity.

**Figure 14:**
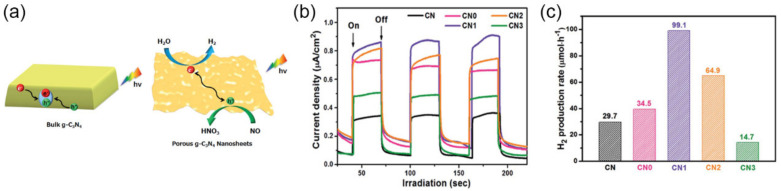
(**a**) Diagram for charge separation of BCN and porous *g*-C_3_N_4_ nanosheets; (**b**) transient photocurrent responses and (**c**) photocatalytic H_2_ evolution rate constants of all samples under visible light (λ > 420 nm), Reprinted with permission from [135] 2012 Royal Society of Chemistry.

Several typical nanostructures of porous *g*-C_3_N_4_ have been applied in water splitting, such as one-dimensional (1D) nanotubes [182,183,184], 2D nanosheets [185,186,187,188], 3D hierarchical porous structure [141,189,190,191,192]. 1D nanostructures are usually arranged in an orderly long range, thus leading to low surface defect density and increased carrier mobility. By using melamine-oxalic acid supramolecules as soft template, porous *g*-C_3_N_4_ nanotubes (length: 200–400 nm; diameter: 100–200 nm) were obtained, labeled as *g*-C_3_N_4_(NT) [193]. *g*-C_3_N_4_(NT) had the average pore diameters of 26.8 nm and the increased SSA of 122.5 m^2^·g^−1^ compared to BCN (10.3 m^2^·g^−1^). Due to the stabilized electron migration along a preferential direction, the decay lifetime of *g*-C_3_N_4_(NT) was prolonged to 27.4 ns from 5.25 ns of BCN, indicating the decreased recombination photoexcited electrons and holes. The H_2_ evolution rate of *g*-C_3_N_4_(NT) was 214.5 μmol·h^−1^, which was about 12-fold higher than BCN (16.8 μmol·h^−1^). Fabrication of 2D nanostructures could not only provide a large SSA, but also enable rapid transfer of photogenerated charge carriers onto the surface of photocatalysts, promoting charge separation [194]. In one case, porous, thin *g*-C_3_N_4_ nanosheets (CN*x*, *x* = 0, 1, 2, 3 presented molar ratios of melamine to thiourea of 1:0, 1:1, 1:3, and 1:6) were obtained by a template-free method which used thiourea-assisted hydrothermal pre-treated melamine as precursor [136]. During the hydrothermal process, thiourea decomposed into H_2_S and NH_3_, resulting in the surface etching and deep peeling of melamine. Thus, after calcination, the porous *g*-C_3_N_4_ nanosheets CN1 not only exhibited the increased SSA (from 9.0 to 44.2 m^2^·g^−1^), but also showed the promoted charge separation (Figure 14a). As proved by the photocurrent response, CN1 had the stronger photocurrent density, which could be up to approximately three times that of BCN (Figure 14b). Therefore, the CN1 showed 3.3-fold increased photocatalytic performance for H_2_ evolution (99.1 μmol·h^−1^), in comparison with BCN (29.7 μmol·h^−1^) (Figure 14c). It should be noted that the ratio between the two precursors affects the pore structure. As in this case, when the thiourea is present in excess, large volumes of gas, this would cause the collapse of the pore structure, which could explain the lower H_2_ evolution rate of CN3 than BCN.

It has been demonstrated that controlling the concentration and distribution of surface vacancy could effectively expand the light response range, tune the energy band structure, and provide more active centers. As shown in one research paper, the carbon vacancies gave porous *g*-C_3_N_4_ a broad light absorption band which even extended into the near-infrared region (800–1400 nm) [195]. Heat treatment of *g*-C_3_N_4_ in the reduced atmosphere, such as H_2_ [196] and NH_3_ [197], can lead to the release of surface atoms and consequently the simultaneous production of homogeneous vacancies on the surface of *g*-C_3_N_4_. For instance, Tu et al., prepared porous *g*-C_3_N_4_ nanosheets with nitrogen vacancies (C/N = 0.66) by heating the BCN to 525 °C in H_2_ atmosphere, named CN-525 [198]. The pores with an average diameter of about 20 nm could be observed on *g*-C_3_N_4_ nanosheets. The total pore volume and the SSA was increased from 0.19 cm^3^·g^−1^ and 21.45 m^2^·g^−1^ of BCN to 0.66 cm^3^·g^−1^ and 87.99 m^2^·g^−1^ of CN-525, respectively. As nitrogen vacancies could cause the excitation of electrons into mid-gap states (−0.688 eV), and the electrons could be more easily excited from VB to mid-gap states than to CB. Relatively, with UV-visible absorption spectra, CN-525 exhibited an extended visible light absorption with a wide shoulder tail (450–700 nm). Therefore, the CN-525 photocatalyst displayed a remarkably higher photocatalytic H_2_ evolution rate of 64.39 μmol·h^−1^, which was around 18 times over BCN.

### 4.2. CO_2_ Reduction

Efficient photocatalytic conversion of CO_2_ is vital to treat CO_2_ emissions and generate beneficial chemicals and fuels. Photocatalytic CO_2_ reduction has extremely complicated photocatalytic reaction mechanisms and pathways involving a proton-assisted multi-electron reduction process with high energy barriers, complex activation, and adsorption of CO_2_ molecules. Due to the higher negative CB potential (−1.4 V), the photogenerated electrons of *g*-C_3_N_4_ works for reducing CO_2_ into various products, such as HCOOH (−0.61 V), CO (−0.53 V), HCHO (−0.48 V), CH_3_OH (−0.38 V), and CH_4_ (−0.24 V), vs. NHE. The pore structure makes *g*-C_3_N_4_ have an advantage in CO_2_ adsorption. In seeking to advance the performance of porous *g*-C_3_N_4_, several approaches have been developed, for example, element doping and formation of heterojunctions with other semiconductors.

**Figure 15:**
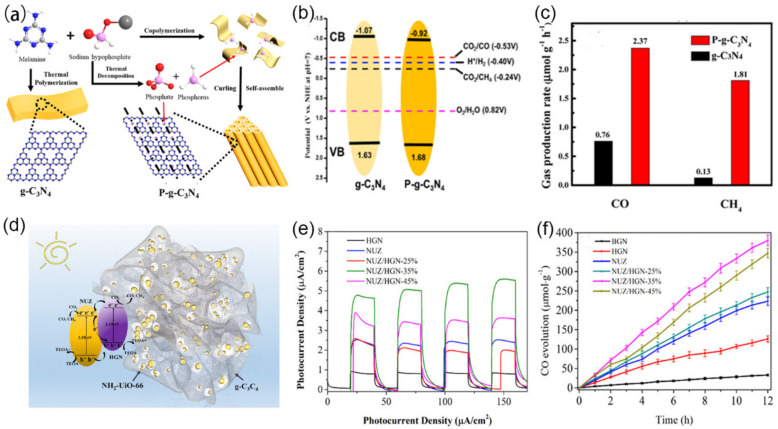
(**a**) Synthesis process of P-*g*-C_3_N_4_; (**b**) the electronic structure and all reaction reduction potentials of CO_2_ conversion into CO and CH_4_; (**c**) gas production rate of P-*g*-C_3_N_4_ and *g*-C_3_N_4_, Reprinted with permission from [206] 2018 American Chemistry Society; (**d**) The diagram of NUZ/HGN composites and diagram for energy band levels of NUZ/HGN composites; (**e**) photocurrent-time curves and (**f**) CO_2_-to-CO conversion performance of HGN, NUZ, and NUZ/HGN under visible light irradiation, Reprinted with permission from [207] 2019 American Chemistry Society.

Heteroatom doping (e.g., B [199], P [200], S [201,202], O [203]) can alter the E_g_, widen the visible light absorption, and boost the charge transfer efficiency to further enhance the photocatalytic CO_2_ conversion properties in the visible-light region [204,205]. Wang et al., reported that CO_2_ reduced to CH_3_OH by the S-doped *g*-C_3_N_4_ photocatalyst with the rate of 1.12 μmol·g^−1^ compared to 0.81 μmol·g^−1^ of un-doped *g*-C_3_N_4_ [202]. In another case, porous P-doped *g*-C_3_N_4_ was obtained through the thermal reaction of melamine and sodium hypophosphite monohydrate (NaH_2_PO_2_·H_2_O) (Figure 15a) [206]. The phosphine gas from the thermal decomposition of NaH_2_PO_2_·H_2_O induced mesopore structure of *g*-C_3_N_4_, giving a pore size of ~25 nm and SSA of 13.38 m^2^·g^−1^. Such mesoporous *g*-C_3_N_4_ showed a narrower E_g_ of 2.58 eV from 2.7 eV (Figure 15b), which was consistent with the wider absorption in 400–550 nm than pristine *g*-C_3_N_4_ (~450 nm). Owing to interstitial doping of P atom, there was an efficient electron-hole separation, as proved by a 2-times photocurrent increase. Therefore, the CO_2_-to-CO and CO_2_-to-CH_4_ conversion rates reached 9.48 and 7.24 μmol·g^−1^ respectively, about 3.12 and 13.9 times higher than those on BCN (Figure 15c).

A series of porous *g*-C_3_N_4_-based heterostructures have been controllably designed and prepared based on the hybridizing strategy. So far, porous *g*-C_3_N_4_ was mainly combined with noble metals, semiconductors, carbons, and polymers, forming binary heterostructures. *g*-C_3_N_4_/semiconductor is a common class of heterogeneous nanostructures used for steering charge kinetics, extending the spectral range for light absorption and enhancing electron-hole separation [208]. OSCs have been widely studied on heterogenization with porous *g*-C_3_N_4_, mainly including dye sensitizers (e.g., porphyrin, phthalocyanine) [209], MOFs, COFs, etc. Especially for organic frameworks, their semiconductor characteristic endows the composite heterostructure for efficient charge transportation, but also crystalline porous structures and extremely large SSA facilitates gas adsorption. Take MOFs as an example, the porous *g*-C_3_N_4_-based composites could achieve a larger surface area (1315.3 m^2^·g^−1^) and strong CO_2_ capture (CO_2_ uptake: 32.7 cm^3^·g^−1^) [210]. In another case, 3D holey *g*-C_3_N_4_ (HGN) was synthesized with melamine-cyanuric acid supramolecular as soft-template and melamine as precursor [207]. The prepared HGN was chemically bonded with NH_2_-UiO-66(Zr) (NUZ) via rich-NHx and Zr-O clusters, yielding NUZ/HGN composites (SSA: 335 m^2^·g^−1^; pore volume: 0.2638 cm^3^·g^−1^) (Figure 15d). As the interfacial interaction would reallocate delocalized electrons among the π-conjugated networks of the HGN and NUZ, the enhancement of light-harvesting capability (200–800 nm) was observed. Besides, the interfacial charge transferring effect efficiently promoted the interfacial separation and migration efficiency of photogenerated charge carriers, as proved by the higher photocurrent density (Figure 15e). However, it was observed that NUZ could not well combine with HGN with the increase in HGN, according to the TEM image (Figure 5f). However, excessive HGN would stagger and overlap in the compound process through van der Waals interactions, which negatively affect the combination of NUZ and HGN and veil the active sites attached by NUZ. As a result, the NUZ/HGN-35% (35 wt.% of HCN) heterojunction showed the highest CO_2_-to-CO conversion rate of 380 μmol·g^−1^ under light illumination for 12 h, which was much higher than that of HGN and NUZ (126 and 203 μmol·g^−1^) (Figure 15f).

### 4.3. Wastewater Treatment

Visible-light-driven wastewater treatment includes photooxidation reaction to degrade organic pollutants or inactivate bacteria, and photoreduction reaction for heavy metals removal. The CB position of *g*-C_3_N_4_ (−1.4 eV) is higher than the superoxide radical (O_2_^−^) production level (O_2_/·O_2_^−^, −0.33 eV vs. NHE) [211], such that the photogenerated electrons can reduce dissolved O_2_ to strongly-oxidizing superoxide radical (O_2_^−^). The generated O_2_^−^ species can react with electrons to form H_2_O_2_. Then, H_2_O_2_ reacts with electrons, forming hydroxyl radicals (OH). Associated with the behavior of the photogenerated carriers and the resonance energy transfer of excitons in polymer *g*-C_3_N_4_, singlet oxygen (^1^O_2_) can be generated [212]. The above reactive oxygen species (O_2_^−^, ^1^O_2_, OH, and H_2_O_2_) generated by *g*-C_3_N_4_ are involved in the of organic pollutants degradation, including organic dyes (MB, MO, RhB, etc.), antibiotics (carbamazepine, tetracycline (TC), etc.), and organic compounds (phenol, oxalic acid, humic acid, etc.). Following a hole-dominant oxidative pathway, bacteria such as *Escherichiacoli* (*E*. *coli*) and *Staphylococcus aureus* (*S*. *aureus*) could be inactivated with an effective and recyclable porous *g*-C_3_N_4_ photocatalyst [213]. Besides photooxidation reaction on porous *g*-C_3_N_4_, the photoreduction for toxic heavy metal ion Cr(VI) or U(VI) has also been investigated [214]. So far, there are many reports that *g*-C_3_N_4_ was used as efficient photocatalysts in wastewater treatment.

**Figure 16:**
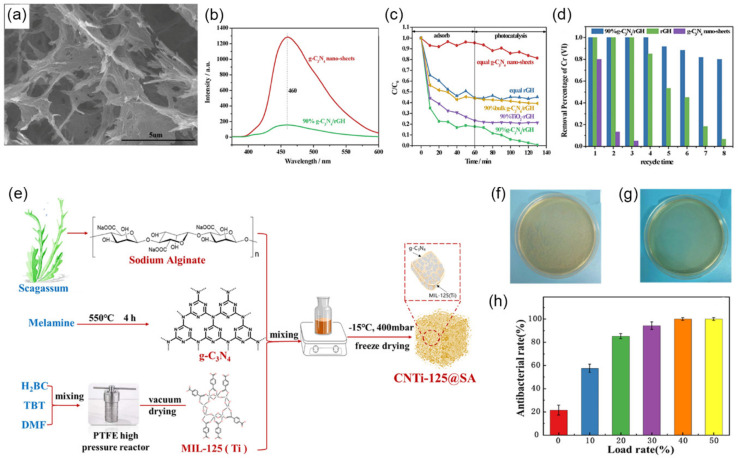
(**a**) SEM image of 90% *g*-C_3_N_4_/rGH; (**b**) PL spectra of *g*-C_3_N_4_ nanosheets, and 90% *g*-C_3_N_4_/rGH; (**c**) Cr(VI) adsorption capacity of different composite materials; (**d**) recycling runs of 90% *g*-C_3_N_4_/rGH in removing Cr(VI) without desorption, Reprinted with permission from [215] 2017 Elsevier; (**e**) Synthesis process of the CNTi-125@SA composite aerogels; antibacterial properties against *E. coli*: (**f**) blank control, (**g**) CNTi-125@SA; (**h**) antibacterial efficiency of the different composite aerogels, Reprinted with permission from [216] 2021 Elsevier.

Recently, developing 3D porous hydrogels or aerogels based on *g*-C_3_N_4_ is a potential approach to improving photocatalysts’ wastewater treatment performance. Keeping *g*-C_3_N_4_ dispersing in gels prevents the stack between the *g*-C_3_N_4_ layers, which forms a highly porous and conductive network. On the one hand, the porous network provides abundant pathways for efficient charge migration, separation of photo-induced carriers, and hole diffusion, and enhances the contact between *g*-C_3_N_4_ and pollutants. On the other hand, the 3D structure makes the catalyst easy to recycle. Previously, several kinds of *g*-C_3_N_4_-based hydrogels or aerogels with enhanced wastewater treatment performance have been reported. Among them, *g*-C_3_N_4_/graphene or *g*-C_3_N_4_/graphene oxide hydrogels are the most widely studied because of the advanced mechanical properties and excellent electronic transmission performance of graphene. Besides, graphene hydrogel can quickly adsorb and enrich Cr ions because of the different modes of pore adsorption and surface adsorption. In one work, *g*-C_3_N_4_ nanosheets/reduced graphene hydrogel (*g*-C_3_N_4_/rGH) system was synthesized [215]. *g*-C_3_N_4_ nanosheets were uniformly dispersed in the 3D gel system, forming the pores with a diameter of about 3.9 nm and some mesopores (12.5–50 nm), giving an SSA of 302.66 m^2^·g^−1^ (Figure 16a). On the one hand, the efficient removal of Cr(VI) is attributed to the high adsorption capacity of the porous network rGH. Results showed that 80% of Cr(VI) (30 mg·L^−1^) could be adsorbed by composite in the dark. However, the photoreduction ability of Cr(VI) was improved because of the decreased recombination of photo-generated electron-hole pairs brought by the excellent electronic transmission performance of reduced graphene. As PL results show in Figure 16b, there was a much lower intensity of *g*-C_3_N_4_/rGH than *g*-C_3_N_4_ nanosheets. Thanks to the synergistic effect of adsorption and photocatalysis, the removal of Cr(VI) reached 100% in 120 min under visible light irradiation via adsorption and photocatalysis (Figure 16c). The rate of treatment was 3.98 and 1.85-fold that of *g*-C_3_N_4_ nanosheets and rGH, respectively. At the same time, this hydrogel system with a micron-level structure was easily recyclable via a stainless-steel filter (pore size: ~38 μm), without the need to rely on a complex separation system, which maintained an 80% removal percentage of Cr(VI) after eight cycles (Figure 16d).

Recently, the utilization of agar, konjac, and sodium alginate (SA) for preparing *g*-C_3_N_4_-based 3D porous gels was considered as a low-cost and eco-friendly strategy to solve the environmental crisis. In the organic pollutant degradation field, *g*-C_3_N_4_ nano-particle was first coated by agar uniformly, forming *g*-C_3_N_4_/agar hybrid hydrogels with a 3D mutual crosslinking network (pore size: 50 μm) by heating-cooling polymerization [217]. Via the synergistic effect of adsorption and photocatalysis, such hydrogels showed 1.3- and 4.5-times improvement of removing phenol and methylene, respectively. In another work, a konjac/*g*-C_3_N_4_ (KCN) aerogel was prepared by freeze-drying the hydrogel of *g*-C_3_N_4_ and konjac [218]. By forming the abundant porous channels (diameter about 200 μm), the O_2_ could be rapidly delivered into the solution, which was a natural electron scavenger. As a result, the concentration of OH and·O_2_^−^ increased, and the oxidation water treatment process was boosted. To remove organic pollutants, the KCN could reach an MB removal of 97.90% and phenol removal of 99.34% in static states. The removal efficiency was kept at 100% in four cycles, indicating excellent cycle stability.

As for the application of antibacterial, Zhao et al., introduced *g*-C_3_N_4_/MIL-125(Ti) heterojunction into sodium alginate (SA), yielding *g*-C_3_N_4_/MIL-125 (Ti) @SA (CNTi-125@SA) composite aerogel after the freeze-drying process (Figure 16e) [216]. Numerous micropores ranging from 50 to 100 μm in size were caused by the astatic growth of ice crystals in the freezing process. The absorbed O_2_ through a large number of pores reacted with MIL-125 (Ti) to generate ·O_2_^−^. Under visible light irradiation, the photogenerated holes were produced on *g*-C_3_N_4_. Organic *E. coli* tended to react with those highly oxidizing substances, eventually producing CO_2_ and H_2_O that completely inactivated the bacteria. The CNTi-125@SA aerogel exhibited an excellent antibacterial efficiency of 100% against *E. coli* (Figure 16f–h).

Photoelectrocatalytic (PEC) oxidation process using a semiconductor photoanode at a small positive potential has proven to be an efficient process for removing various pollutants from water. *g*-C_3_N_4_ has been recognized as promising for organics degradation, particularly in PEC application [219,220]. In one case, Liang et al., applied *g*-C_3_N_4_ electrode as the photoanode for phenol degradation [221]. Under visible light irradiation, the phenol could be depleted completely by the *g*-C_3_N_4_ with a 2.5 V bias, and 89.3% of the total organic carbon was removed. Recently, researchers utilized the PEC oxidation process of *g*-C_3_N_4_ to decompose noble metal-cyanide complexes and simultaneously recover noble metals from wastewaters [222]. A peroxymonosulfate/*g*-C_3_N_4_ nanosheet composites resulted in high Ag-cyanide decomposition and Ag recovery efficiencies (74% and 68%, respectively) within 150 min under visible light [223]. Semiconductors-based PEC technique has been proved to be an effective way to generate a synergistic effect for achieving H_2_ evolution and organic pollutant degradation. The mechanistic pathway is that photogenerated electrons are transferred to the cathode for H_2_ evolution under applied bias voltage, while organic compounds are degraded in the anode [224,225].

## 5. Conclusions

In general, *g*-C_3_N_4_, as an excellent photocatalyst, has a lot of development space in the field of photocatalysis in the future. *g*-C_3_N_4_ itself is non-toxic, pollution-free, and environmentally friendly. Pore structure endows *g*-C_3_N_4_ with a high SSA to provide more active sites, enhancing light absorption ability, photogenerated charges separation and transfer, and mass transfer for a redox reaction, thus greatly improving the photocatalytic performance. The construction and modification methods of *g*-C_3_N_4_ with a large SSA coordination pore structure have all been extensively researched in various photocatalytic applications. In this review, we first discussed the relationship between pore structure and photoelectric performance. We then reviewed three pore-modifying methods (hard-template, soft-template, and template-free methods) in detail. The advantages and disadvantages of each preparation method were emphasized and compared. Moreover, we also introduced recent achievements in pore structure *g*-C_3_N_4_ with the commonly used modification methods in photocatalytic fields, including water splitting, CO_2_ reduction, and wastewater treatment. Overall, porous *g*-C_3_N_4_ as an excellent photocatalyst has a huge development space in photocatalysis in the future.

Although great progress has been made in researching porous *g*-C_3_N_4_ photocatalysts, several challenges remain. The improvement direction of the hard-template method is to simplify the preparation process and make it pollution-free. The research of the pore-modifying method transfers from hard-template method to soft-template method and template-free method because of the advantages, such as simple equipment, easy operation, and low cost. As for the soft-template and template-free methods, future development needs to pay more attention to designing the experimental scheme and selecting the precursors to alleviate interlayer collapse, interlayer stacking, and molecular polymerization to increase the SSA of the prepared samples. In addition, suitable functional modification methods also play a significant role in tuning energy band structure and constructing interfacial electron transfer. However, the effect of the second component on the pore structure of *g*-C_3_N_4_ should be considered. Additionally, it is of great significance to develop large-scale applications of *g*-C_3_N_4_ photocatalysts in practical use. Since the performance of photocatalysts will be greatly reduced in real uncontrolled conditions and the deactivation will be faster in real environments, the performance testing conditions need to be more specific.

## Figures and Tables

**Table 1:** Summary of the different template methods in adjusting the pore structure of *g*-C_3_N_4_ and their photocatalytic performance.

Sample	Template	Removal Reagents	Pore Size (nm)	Pore Volume (cm^3^·g^−1^)	SSA (m^2^·g^−1^)	Photocatalytic Application (Efficiency)	References
Hard-template method							
Meso-*g*-C_3_N_4_/WP/Meso-*g*-C_3_N_4_	SiO_2_	HF	12	-	82	H_2_ evolution (198.1 μmol·h^−^^1^·g^−^^1^)	[171]
Porous-C_3_N_4_	SiO_2_	HF	5–40	-	209	MB degradation (85% in 180 min)	[85]
mp*g*-C_3_N_4-δ_	SiO_2_	NH_4_HF_2_	12.54	0.69	218.15	RhB degradation (30.2% in 30 min)	[172]
mesoporous *g*-C3N4	SiO_2_	NH_4_HF_2_	12	0.52	190.7	U(VI) reduction (73% in 60 min)	[87]
TiO_2_ trapped *g*-C_3_N_4_	PSB	NH_4_HF_2_	27	0.30	37	RhB degradation (100% in 50 min)	[173]
CN-MCF-0.4	MCF	NH_4_HF_2_	5.3	1.36	498	Knoevenagel condensation (93.6% in 240 min)	[174]
C_3_N_4_-MCF	MCF	HF	5/20	0.30	70	CO_2_ reduction (8.0 μmol·g^1^)	[94]
*g*-C_3_N_4_(GLBM-SBA-15)	SBA-15	NH_4_HF_2_	11/90	0.43	145	MO degradation (90% in 30 min)	[83]
macrostructure *g*-C_3_N_4_	NaCl	H_2_O	-	-	16.71	H_2_ evolution (459 μmol·h^1^·g^−^^1^)	[109]
Na^+^ functionalized porous *g*-C_3_N_4_ nanorods	Na_2_S_2_O_3_	H_2_O	4.25	-	29.72	H_2_ evolution (9796 μmol·h^1^·g^−^^1^)	[103]
Na_2_CO_3_-embedded porous crystalline *g*-C_3_N_4_	NaHCO_3_	H_2_O	3/30	0.09	15.34	H_2_ evolution (1010 μmol·h^1^·g^1^)	[175]
polymeric CN nanocages	ZnO	HCl and NH_3_·H_2_O	16.8	0.103	32	H_2_ evolution (227.23 μmol·h^1^·g^−^^1^)	[176]
Soft-template method							
*g*-C_3_N_4_	P123	-	20	-	90	H_2_ evolution (60.6 μmol·h^1^)	[123]
3D mesoporous CN	ionic liquid	-	15	0.85	381	H_2_ evolution (129.5 μmol·h^−^^1^)	[177]
hollow mesoporous *g*-C_3_N_4_ sphere	ionic liquid	-	9.7	-	84	H_2_ evolution (157 μmol·h^−^^1^)	[28]
*g*-C_3_N_4_/SnO_2_	HCl, H_2_O	-	100/430	2.638	44.3	MB degradation (72% in 100 min)	[127]
porous *g*-C_3_N_4_ nanosheets	NH_3_, CO_2_	-	5/70	-	92.75	H_2_ evolution (1437 μmol·h^1^·g^−^^1^)	[178]
Macroscopic 3D porous *g*-C_3_N_4_ monolith	melamine	-	4/30–170	0.76	78	H_2_ evolution (29 μmol·h^−^^1^)	[30]
Template-free method							
Thin-layered *g*-C_3_N_4_ nanosheets	-	-	4/20–100	0.752	92.8	H_2_ evolution (1391 mmol·h^1^·g^−^^1^)	[86]
*g*-C_3_N_4_/TiO_2_	-	-	11	0.193	70.2	RhB degradation (0.0478 min^−^^1^)	[80]
S-doped CN	-	-	17.04	0.464	108.9	H_2_ evolution (567.7 μmol·h^1^·g^−^^1^)	[179]
oxygen-doped *g*-C_3_N_4_ nanosheets	-	-	5–100	-	-	H_2_ evolution (189.3 μmol·h^−^^1^)	[180]
Porous *g*-C_3_N_4_ nanosheets	-	-	5–25	0.61	190.1	MB degradation (44.7% in 30 min)	[181]
mp*g*-C_3_N_4_/rGO	-	-	10–80	0.419	85	RhB degradation (99.7% in 40 min)	[61]
porous *g*-C_3_N_4_	-	-	4.5/40.5	0.211	44.2	H_2_ evolution (99.1 μmol·h^−^^1^)	[135]

## Data Availability

Not applicable.
